# One-Step Synthesis of Green Fluorescent Carbon Dots for Chloride Detecting and for Bioimaging

**DOI:** 10.3389/fchem.2021.718856

**Published:** 2021-09-17

**Authors:** Juan Yue, Ling Yu, Li Li, Pai Liu, Qian Mei, Wen-Fei Dong, Ru Yang

**Affiliations:** ^1^ Division of Life Sciences and Medicine, School of Biomedical Engineering (Suzhou), University of Science and Technology of China, Hefei, China; ^2^ The Affiliated Suzhou Hospital of Nanjing Medical University, Suzhou, China; ^3^ CAS Key Laboratory of Biomedical Diagnostics, Suzhou Institute of Biomedical Engineering and Technology, Chinese Academy of Science (CAS), Suzhou, China; ^4^ Jinan Guokeyigong Science and Technology Development Co., Ltd, Jinan, China

**Keywords:** carbon dots, chloride ion, fluorescence sensing, off–on, bioimaging.

## Abstract

The chloride ion is an essential ion in organisms, which plays an important role in maintaining normal cell functions. It is involved in many cell activities, such as cell proliferation, cell excitability regulation, immune response, and volume regulation. Accurate detection of the chloride ion can balance its concentration *in vivo*, which is of great significance. In this study, we developed a green fluorescent carbon quantum dot to detect chloride concentration through the “off–on” mechanism. First, the fluorescence of carbon dots is quenched by the complex of sulfhydryl and silver ions on the surface of carbon dots. Then, the addition of chloride ions pulls away the silver ions and restores the fluorescence. The fluorescence recovery is linearly related to the concentration of chloride ions, and the limit of detection is 2.817 μM, which is much lower than those of other reported chloride probes. Besides, cell and zebrafish experiments confirmed the biosafety and biocompatibility of the carbon dots, which provided a possibility for further applications in bioimaging *in vivo*.

## Introduction

Chloride (Cl^−^), one of the most abundant anions in the extracellular fluid (ECF), accounts for about 80% of the total ions that maintain osmotic pressure combining with Na^+^, which plays crucial roles in regulation of ECF volume and maintaining osmotic pressure (Doyon et al., 2016; Ke, 2020). Cl^−^ is closely related to maintaining the acid–base balance of body fluids. Moreover, Cl^−^ participates in the process of cell proliferation, regulation, excitability, and immune response, and more importantly, it can stabilize cell membrane potential (Cuartero et al., 2018; Zhang et al., 2020). If the chloride ion is out of balance, it will cause serious diseases, such as cystic muscular dystrophy, sickle cell anemia, fibrosis, and so on (De Koninck, 2007; Kaila et al., 2014; Zajac et al., 2020). In addition, in the field of chemical synthesis, halide ions can also be used to synthesize and control the morphology and structure of nanocrystals (Lv et al., 2021). Therefore, efficient and sensitive detection for Cl^−^ is necessary. At present, there are many methods to detect Cl^−^, such as ion-sensitive field effect transistors (ISFETs) (Bratov et al., 2004), ion chromatography (IC) (Lopez-Moreno et al., 2016; Robaina et al., 2016), microfluidic devices (Chou et al., 2012), and electrochemical methods (Chu and Zhang, 2012; Rahman et al., 2013; Bujes-Garrido and Arcos-Martínez, 2016; Bujes-Garrido and Arcos-Martínez, 2017). Unfortunately, these methods either require expensive instruments and tedious operations or have low detection accuracy and poor specificity. Due to the strong chemical stability and inert chemical reaction of the chloride ion, there are only a few methods for the detection of the chloride ion based on chemiluminescence and fluorescence spectroscopy (Kim et al., 2017). For example, Han developed a fluorescent probe for the chloride ion using Ag-benzimidazole complexes (Kim et al., 2020), and Bazany-Rodríguez et al. (2015) reported a water-soluble fluorescent probe for chloride based on a bisquinolinium pyridine-dicarboxamide compound. However, organic fluorescent probes have the disadvantages of poor stability and low fluorescence intensity, so it is necessary to develop chloride fluorescent probes based on nanomaterials.

Carbon dots (CDs), a kind of nanoparticles with a particle size less than 10 nm, have the advantages of good biocompatibility, high water solubility, a high fluorescence quantum yield, strong anti-bleaching ability, and an adjustable emission wavelength (Liu et al., 2019b; Qu et al., 2019; Qu and Sun, 2020; Yang et al., 2020). By improving the synthesis method or surface modification, CDs have been widely used in the detection of ions or small molecules (Liu et al., 2019a; Gong et al., 2019; Qi et al., 2019; Hu et al., 2021). Yang et al. reported the detection of Fe^3+^ with blue fluorescent carbon dots and investigated its detection mechanism (Song et al., 2014). Li et al. realized the detection of the calcium ion by carbon dot-modified Ethylenebis(oxyethylenenitrilo)tetraacetic acid (EGTA) (Yue et al., 2019). Cao et al. used carbon dots to detect progesterone concentration based on the oxidation–reduction mechanism (Cao et al., 2020). Also, Qin et al. reported a dually emitting carbon dot as a fluorescent probe for ratiometric fluorescence sensing of pH values, mercury (II), and chloride (Li et al., 2019).

**SCHEME 1:**
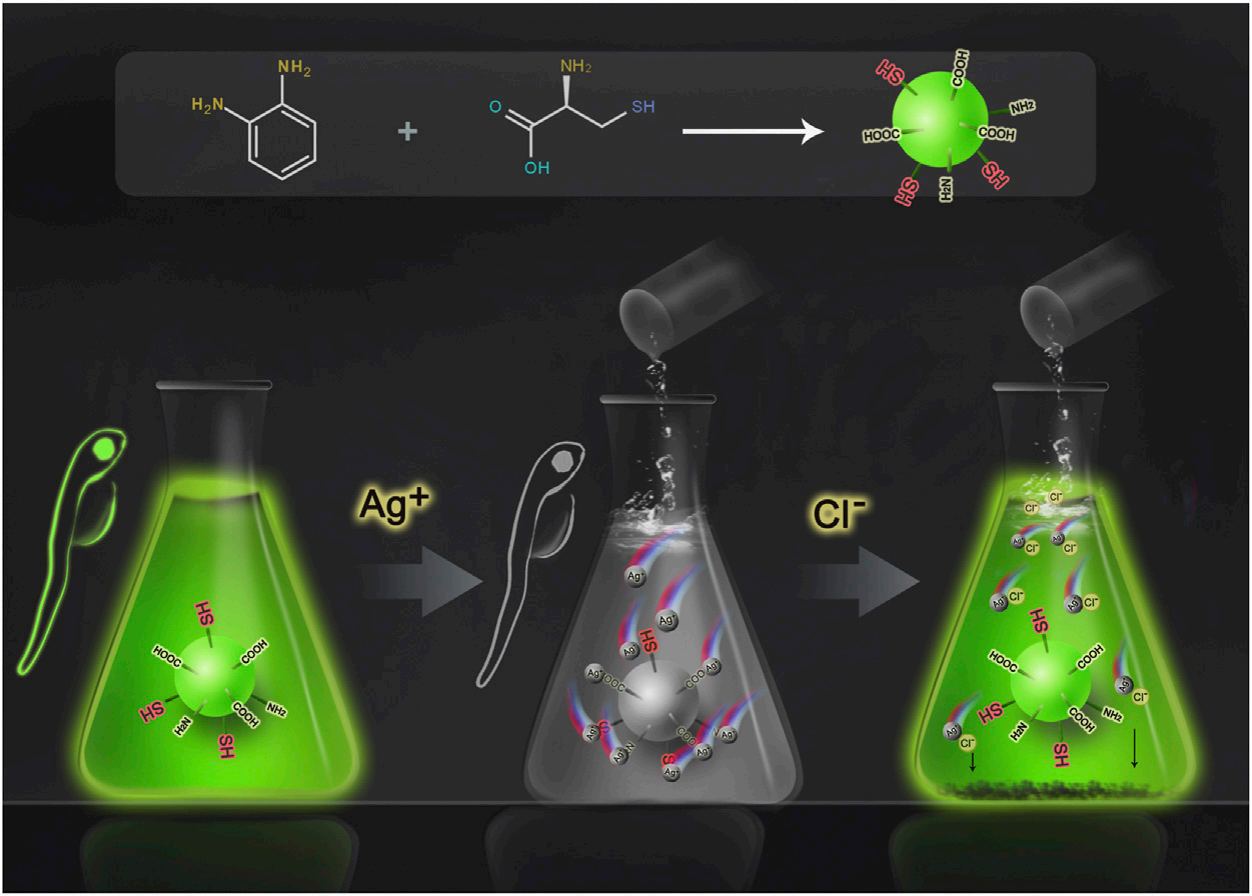
Schematic diagram of Cl^−^ detection by CDs.

Herein, we propose to use carbon dots to detect chloride ions based on the “off–on” fluorescence process (Scheme 1) (Barman and Sadhukhan, 2012; Zheng et al., 2013). The carbon dots prepared from o-phenylenediamine and L-cysteine showed bright green fluorescence with a quantum yield (QY) of 0.14, and the surface was rich in carboxyl, amino, and thiol groups. These groups can easily capture Ag^+^ ions and lead to the quenching of the fluorescence of carbon dots. After the addition of Cl^−^ ions, the Ag^+^ ions are separated from the surface of carbon dots due to the stronger binding ability of Ag^+^ with Cl^−^; thus, the green fluorescence of carbon dots is restored. The experimental results show that in the range of 15–200 μM, the fluorescence recovery of CDs is linearly related to the concentration of chloride ions, and the limit of detection (LOD) is as low as 2.817 μM, which is much lower than that of other reported sensors or probes, whose LOD is at least 19 μM (for specific performance comparison, see Supplementary Table S1). At the same time, cellular experiments confirmed that the CDs were non-toxic and harmless and had good biocompatibility. Finally, fluorescence imaging in live zebrafish was achieved using the CDs as fluorescent dyes. In general, the CDs can be used as an effective chloride ion fluorescence probe and have a good application prospect.

## Experimental

### Chemicals and Materials

All reagents, such as o-phenylenediamine, L-cysteine, silver nitrate, sodium chloride, and so on, were commercially obtained from Aladdin Reagent Co., Ltd. (Shanghai, China).

#### Zebrafish

Zebrafish were purchased from Hangzhou Huante Biological Technology Co., Ltd. They were cultured in a laboratory circulating aquaculture system with the cycle of 14 h of light and 10 h of darkness at a temperature of 28 ± 0.5°C. We selected two male and two female zebrafish into different spawning boxes the day before the experiment. Then, the male and female were mixed under light conditions the next day, and the eggs were collected 1 h later.

### Preparation of CDs

According to the literature, CDs are synthesized by the hydrothermal method and modified. Briefly, 0.5 g of L-cysteine and 0.5 g of o-phenylenediamine were completely dissolved in 30 ml of deionized water; then, the mixture was added to a 50 ml Teflon-equipped stainless-steel autoclave and heated at 180°C for 8 h. After cooling to room temperature, the products were then placed in a dialysis membrane (molecular weight cutoff 500), and the rest of the reaction material was removed. Finally, the CDs were obtained by vacuum freeze drying for further use.

### Characterization of CDs

Transmission electron microscopy (TEM) (JEOL Ltd, Japan) was used to analyze the morphology of CDs. A Nano ZS/ZEN3690 (Malvern, UK) was employed to investigate the particle size distribution. The Fourier transform infrared spectroscopy (FT-IR) spectra were recorded from a Cary 660 FT-IR Spectrometer (Agilent, USA). The UV absorption spectra and the fluorescent spectra were respectively recorded using a U-3900 spectrophotometer (Hitachi, Japan) and the fluorescence spectrophotometer F-4600 (Hitachi, Japan). A Kratos AXIS Ultra DLD X-ray Photoelectron Spectrometer (Shimadzu, Japan) was used to analyze the surface characterization.

### QY Calculation

Using quinine sulfate as the standard control, the fluorescence QY of CDs was calculated according to the following formula:

$$Q_{C}=Q_{R}\cdot \frac{I_{C}}{I_{R}}\cdot \frac{A_{R}}{A_{C}}\cdot \frac{\eta_{C}^{2}}{\eta_{R}^{2}},$$

where “*Q*” is the quantum yield, “*I*” is the integral fluorescence intensity, “*η*” is the refractive index of the solvent, and “*A*” is the absorbance. Subscripts “*C*” and “*R*” represent CDs and quinine sulfate, respectively.

### Detection of Cl^−^

Cl^−^ was determined by a two-step method. At first, we added AgNO_3_ aqueous solution to the CD aqueous solution to observe the changes of fluorescence intensity of CDs by titration. Briefly, AgNO_3_ aqueous solution was added to the CD (10 μg/ml) aqueous solution with a working concentration range from 50 to 400 μM. Following on, NaCl aqueous solution (200–400 μM) was added to the above mixed solution to detect the fluorescence intensity of CDs.

### Cell Cytotoxicity Assay and Endocytosis

The 3-(4,5-dimethylthiazol-2-yl)-2,5-diphenyltetrazolium bromide (MTT) method is used to detect the cytotoxicity of CDs. 4T1 cells were seeded in 96-well plates with a density of 2 × 10^3^ cells per well. Then, different concentrations of CDs (50~300 μg/ml) were incubated with the cells for 24 h. The supernatant was removed, and the cells were washed with phosphate-buffered saline (PBS). Then, each well was injected with 20 μL of MTT (5 mg/ml) and incubated again for 4 h. Finally, the absorbance was detected by a microplate reader, and the cell viability of each treatment group was calculated.

4T1 cells were seeded into cell dishes at a density of 2 × 10^4^ cells per well with Dulbecco’s modified Eagle’s medium containing 10% fetal bovine serum (FBS). CDs at a concentration of 30 μg/ml were added into the 4T1 cells and co-incubated for 24 h. The cells were then washed with PBS and observed using confocal scanning microscopy (CLSM).

### Fluorescence Imaging of Live Zebrafish

Here, we used CDs as a probe to observe fluorescence in live zebrafish. Introducing CDs into zebrafish embryos, larvae, and full-grown zebrafish by soaking was described briefly. A total of 50 normally developed embryos and 20 larvae were soaked in an aqueous solution with CDs (0.5 mg/ml) for 24 h. Then, these embryos and larvae were washed six times to remove the CDs on the surface, and a fluorescence microscope was used to analyze biological fluorescence imaging of CDs.

## Results and Discussion

### Characterizations of CDs

**FIGURE 1:**
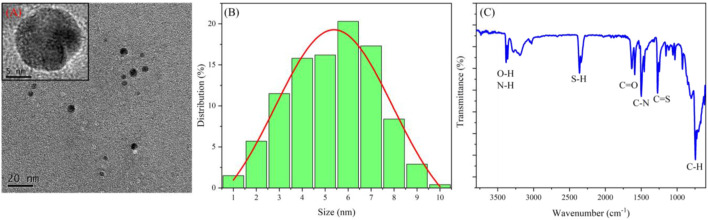
**(A)** TEM images of CDs; **(B)** size distribution of CDs; **(C)** FT-IR spectrum of CDs.

TEM and dynamic light scattering (DLS) characterized the morphology and size of CDs, respectively. From the TEM image (Figure 1A), the CDs are well dispersed, showing a uniform spherical shape with a size of about 3–8 nm. Also, the high-resolution TEM image clearly shows that the CDs have a high-resolution lattice structure corresponding to the graphite structure. The DLS result proves again that the diameter of CDs is less than 10 nm, and most of them are in the range of 6–7 nm (Figure 1B).

FT-IR was used to study the properties of surface groups of CDs (Figure 1C). The peak at 3,470 cm^−1^ corresponds to the vibration of O–H and N–H; the peaks at 2,400, 1,640, and 1,500 cm^−1^ are respectively attributed to S–H, C=O, and C–N bonds (Hsiao et al., 2017; Nikoorazm et al., 2018; Shi et al., 2018); also, C=S and C–H bonds bring peaks at 1,380 and 900 cm^−1^, respectively. In general, FT-IR results show that the surface of CDs is rich in hydroxyl, carbonyl, amino, and thiol groups.

**FIGURE 2:**
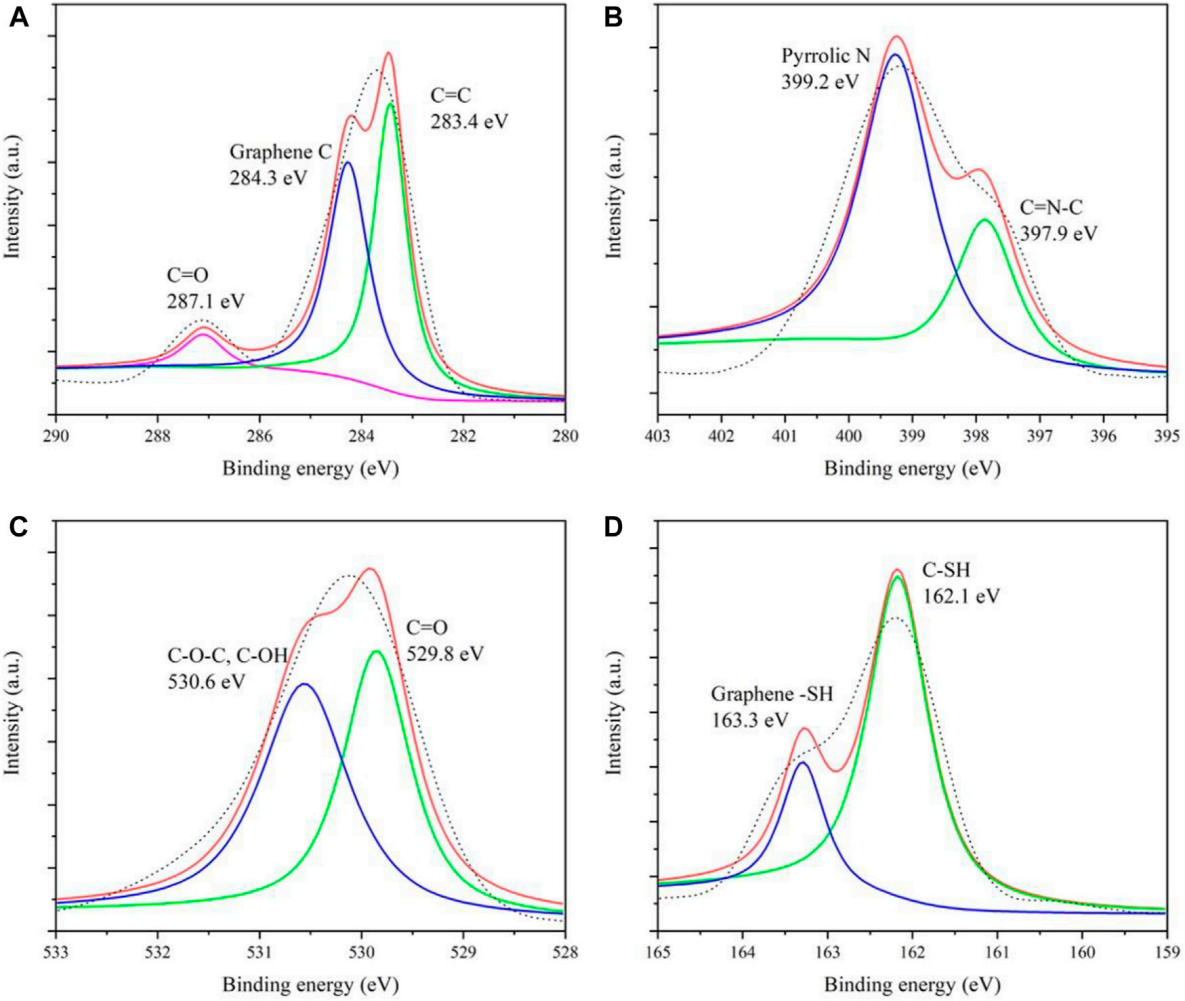
High-resolution XPS spectra of C 1s **(A)**, N 1s **(B)**, O 1s **(C)**, and S 2p **(D)**.

In addition, the elements and chemical bonds of CDs were further investigated by the X-ray photoelectron spectrometry (XPS) test. Supplementary Figure S1 gives four main peaks, located at 283.7, 399.4, 529.8, and 163.2 eV, corresponding to C 1s, N 1s, O 1s, and S 2p, respectively. Figure 2A manifests that the spectral envelope of C 1s could be de-convoluted into three peaks, 287.1, 284.3, and 283.4 eV, respectively assigned to C=O, C–N/C–O, and C–C/C=C. Figure 2B gives two peaks of N 1s at 399.2 eV (N–H) and 397.9 eV (N–C), proving the presence of a graphite-like structure and NH_2_ group, respectively. Also, for O 1s (Figure 2C), there are two high peaks at 530.6 (C–O) and 529.8 eV (C=O) due to the presence of –OH and C=O on the CD surface. In the meantime, the high-resolution spectra of S 2p (Figure 2D) composed of two peaks at 163.3 and 162.1 eV certified that the CDs contain the graphene–SH and C–SH clearly. XPS has confirmed that the CDs have hydroxyl, carbonyl, amino, and sulfhydryl groups, which is why the CDs can easily complex Ag^+^ ions.

**FIGURE 3:**
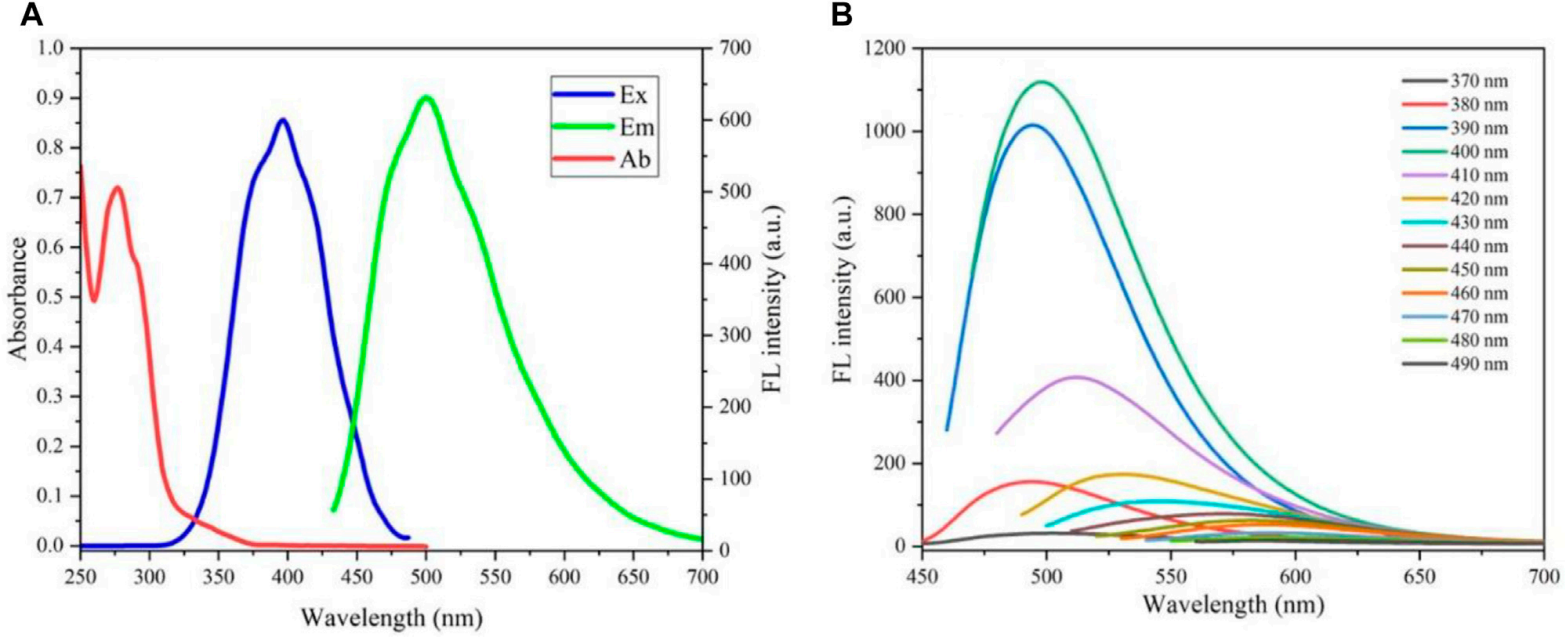
**(A)** UV–vis absorption (red line), excitation (blue line), and photoluminescence emission (green line) spectra of CDs; **(B)** fluorescence spectra under different excitations.

Then, the absorption and fluorescence properties of CDs were tested. Figure 3A shows a strong absorption peak at 280 nm from the intrinsic absorption of the benzene ring and its derivatives. Also, with 400 nm excitation, the optimal emission wavelength of the CDs is located at 501 nm. Upon different excitation wavelengths (from 370 to 490 nm), the fluorescence reveals excitation-dependent characteristics (Figure 3B). When the excitation wavelength is lower than 380 nm, the fluorescence intensity is weak; upon excitation between 390 and 400 nm, the CDs have the strongest fluorescence, and when the excitation wavelength is higher than 410 nm, as the excitation wavelength increases, the emission wavelength red-shifts, but the intensity decreases sharply. Therefore, in this work, all the tests are completed under the fluorescence emission of 501 nm with the excitation wavelength of 400 nm. In addition, the prepared CDs have fluorescence stability under different pH conditions (Supplementary Figure S2A). Moreover, after 10 days of storage at room temperature, the fluorescence intensity of CDs only decreased by 10% (Supplementary Figure S2B).

### Detection Cl^−^ in Aqueous Solutions

**FIGURE 4:**
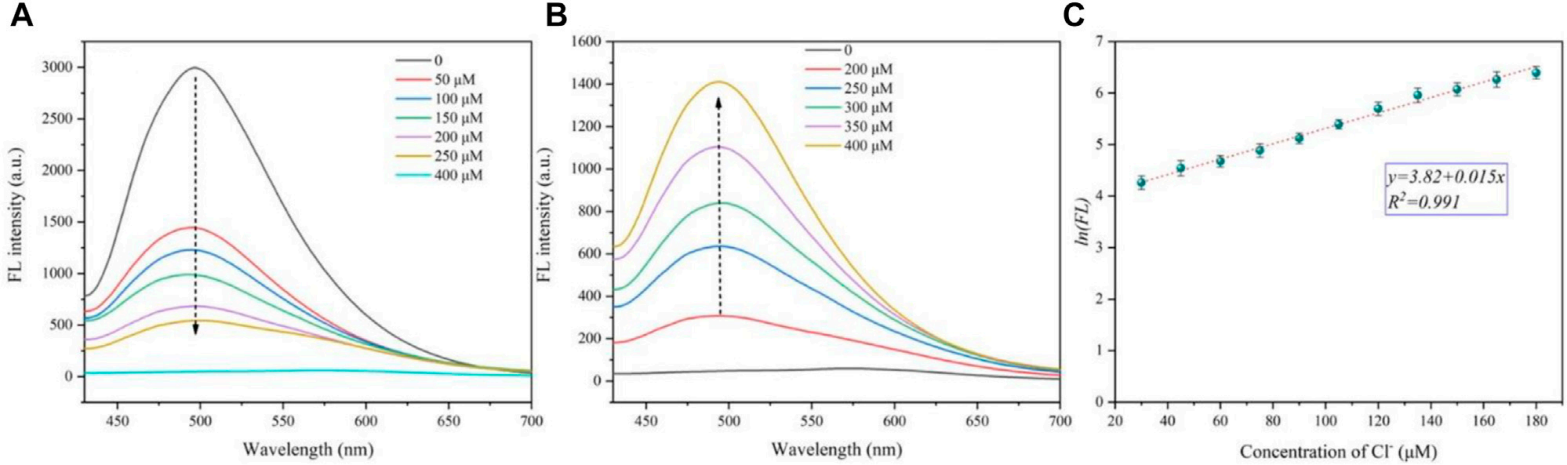
**(A)** Fluorescence decrease curves of CDs (10 μg/ml) with different Ag^+^ concentrations (from 0 to 400 μM). **(B)** Fluorescence recovery curves of CDs (10 μg/ml) coating Ag^+^ (400 μM) with different Cl^−^ concentrations (from 0 to 400 μM). **(C)** Linear relationship between the natural logarithm of fluorescence intensity and chloride concentration.

The detection of the chloride ion by CDs is realized by a two-step method. First, the fluorescence of the CDs is quenched by silver ions. As shown in Figure 4A, with the increase of silver ion concentration, the fluorescence intensity of CDs at 501 nm decreases sharply, and when the silver ion concentration reaches 400 μΜ, the fluorescence intensity of CDs is almost completely extinguished and the position of fluorescence wavelength remains unchanged. Second, we explored the effect of Cl^−^ on CD fluorescence on the basis of the above experiment. With the addition of chloride ions into the CD solution with Ag^+^, the fluorescence intensity of the solution at 501 nm gradually recovers (Figure 4B), and after adding 400 μM of chloride ions, the fluorescence intensity reaches 47% of the original CD solution.

Furthermore, the detection limit of the chloride ion was calculated with a low concentration of CDs (5 μg/ml) (see Supplementary Figure S3 and Supplementary Table S2 for calculation details). The natural logarithm of fluorescence intensity (y) is found to be well linear (y = 3.82 + 0.015x, *R* ^2^ = 0.991) versus the Cl^−^concentration ranging from 30 to 180 μΜ (Figure 4C). The LOD of Cl^−^ is calculated to be 2.817 μM. Therefore, carbon dots are considered as a good fluorescent “off–on” probe, which can detect the concentration of chloride ions in a certain range.

### “Off–On” Mechanism of the CDs for Ag^+^ and Cl^−^

**FIGURE 5:**
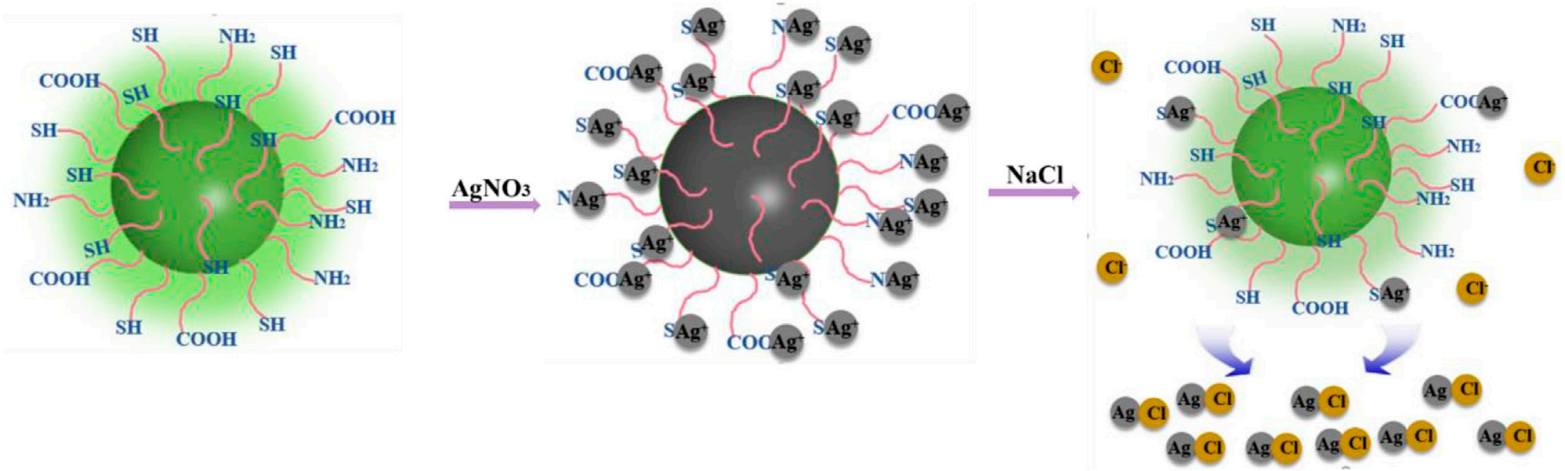
Schematic diagram of CD fluorescence quenching and recovery.

The “off–on” mechanism of the CDs for Ag^+^ and Cl^−^ is summarized in Figure 5. The CDs rich in sulfhydryl, carboxyl, and amino groups exhibit bright green fluorescence. When Ag^+^ ions are added, the functional groups of CDs are complexed with Ag^+^ ions, turning off the green fluorescence. After that, with the addition of Cl^−^ ions, due to the stronger binding ability of Cl^−^ ions and Ag^+^ ions, Ag^+^ ions were separated from the surface of CDs, and the CDs returned to the monodisperse free state, resulting in the recovery of fluorescence. Generally, this is a competitive reaction between silver ions and anions. The combination and separation of silver ions and carbon dots bring about the “off–on” fluorescence of CDs, thus realizing the detection of chloride ions.

### Selectivity of Cl^−^ Detection

**FIGURE 6:**
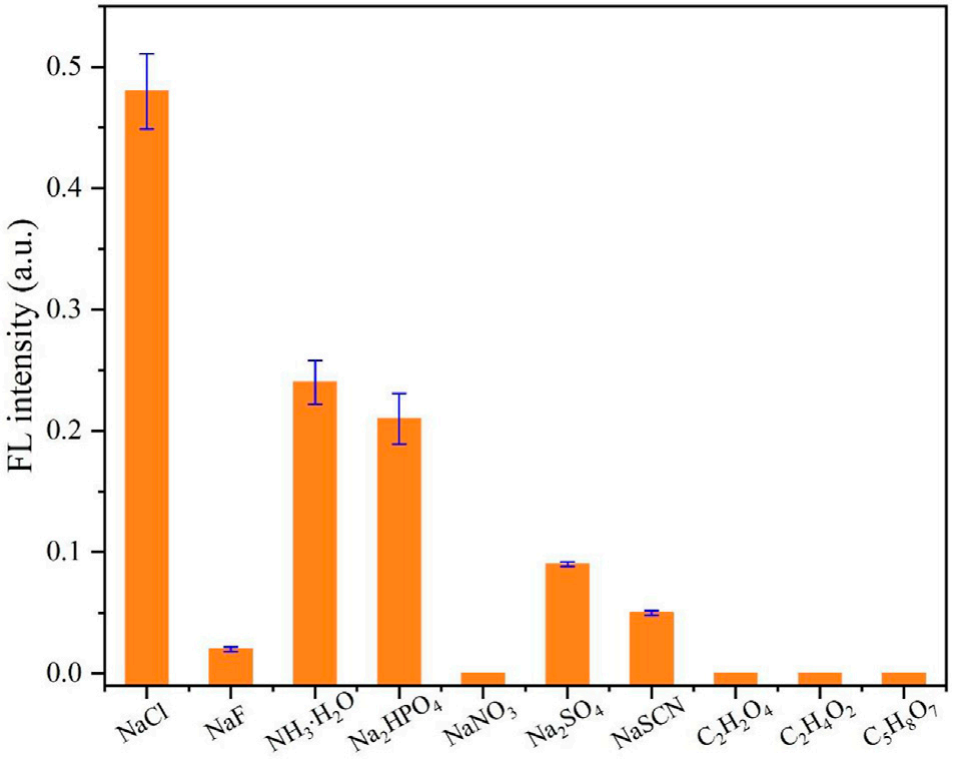
Selectivity test toward Cl^−^ using CDs (10 μg/ml) with Ag^+^ (400 μM). The concentration of competitive anions or molecules is 400 μM.

In order to test the influence of other ions and small molecules on the detection of chloride ions, interference experiments were carried out. As illustrated in Figure 6, the fluorescence of CDs with Ag^+^ will not recover obviously when different anions or molecules including F^−^, NO^3-^, SO_4_^2-^, SCN^−^, C_2_H_2_O_4_ (oxalic acid), C_2_H_4_O_2_ (acetic acid), and C_5_H_8_O_7_ (citric acid) are added. Also, after the addition of NH₃·H₂O or HPO_4_^2-^, the fluorescence intensity is increased slightly. We think that although the binding ability of the sulfhydryl group to the silver ion is stronger than that of ammonia and phosphate, the binding is a dynamic equilibrium process; that is, there are both complexing and dissociating between the silver ion and sulfhydryl group on the surface of CDs in the solution (usually, the complexing trend is much greater). Also, when the ammonium ion and phosphate ion in the solution reach a certain level, the ammonium ion or phosphate ion will also complex with the silver ion, resulting in the partial recovery of the fluorescence. However, these two anions do not interfere with the detection of chloride ions by CDs because chloride ions can enhance fluorescence by about 50%, which is much larger when compared to other ions. It can be considered that the selectivity of CDs to the chloride ion mainly comes from the large stability constant between the chloride ion and silver ion. Although it is well known that the stability constant of the sulfhydryl group with the silver ion is much greater than that of the chloride ion, the silver ion combined with sulfhydryl group will be difficult to obtain by the chloride ion. However, the surface of the prepared CDs is rich in other ions, such as carboxyl, amino, and hydroxyl groups, and the content of the sulfhydryl group is only a small part. Therefore, when the chloride ion is excessive, the silver ion can be pulled away from the CDs so that the fluorescence of CDs can be restored.

### Cytotoxicity and Endocytosis of CDs

**FIGURE 7:**
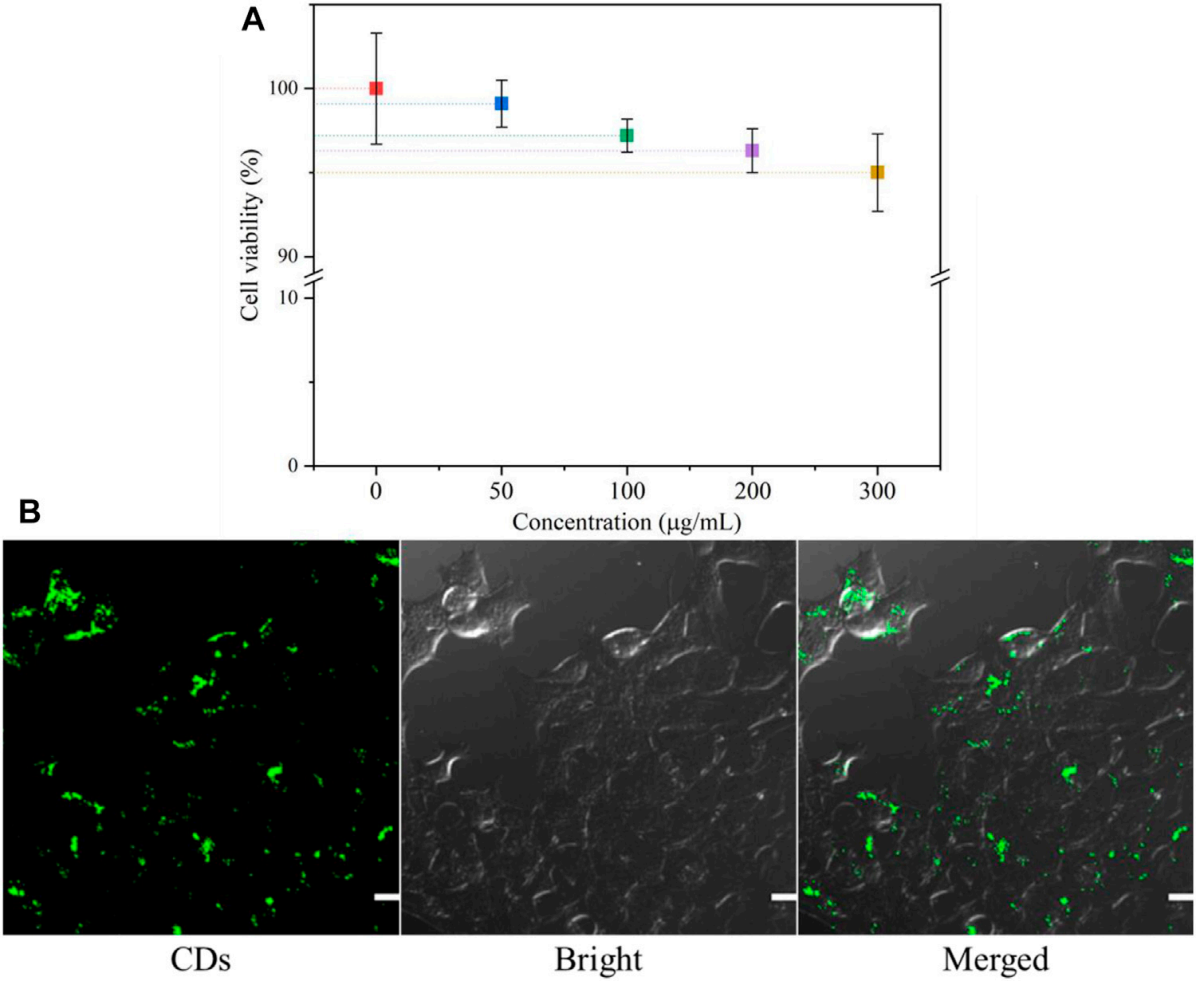
**(A)** MTT assay results for the viability of 4T1 cells in different concentrations of CDs; **(B)** CLSM images of 4T1 cells co-incubated with CDs for 24 h. The scale bar is 50 μm.

To assess the cytotoxicity of the CDs *in vitro*, the cell viability of 4T1 cells was tested by the MTT assay. Figure 7A shows that the cell viability is still higher than 95% even at a concentration of 300 mg/ml CDs, indicating that the CDs are almost non-toxic to cells and have good biocompatibility. Generally, the concentration of carbon dots used in biological experiments will not exceed 100 mg/ml. Thus, the CDs have potential applications in fluorescence imaging of cells or animals.

In order to better explore the potential biomedical applications of the CDs, it is critical to determine their cellular uptake activity. 4T1 cells were incubated with 30 μg/ml CDs for 24 h, and their endocytosis was measured by confocal microscopy. As shown in Figure 7B, cells treated with the CD solution showed bright green fluorescence, suggesting that most CDs had entered the cells. The result indicated that CDs may be a useful probe for intracellular detection.

### Fluorescence Imaging of Zebrafish

**FIGURE 8:**
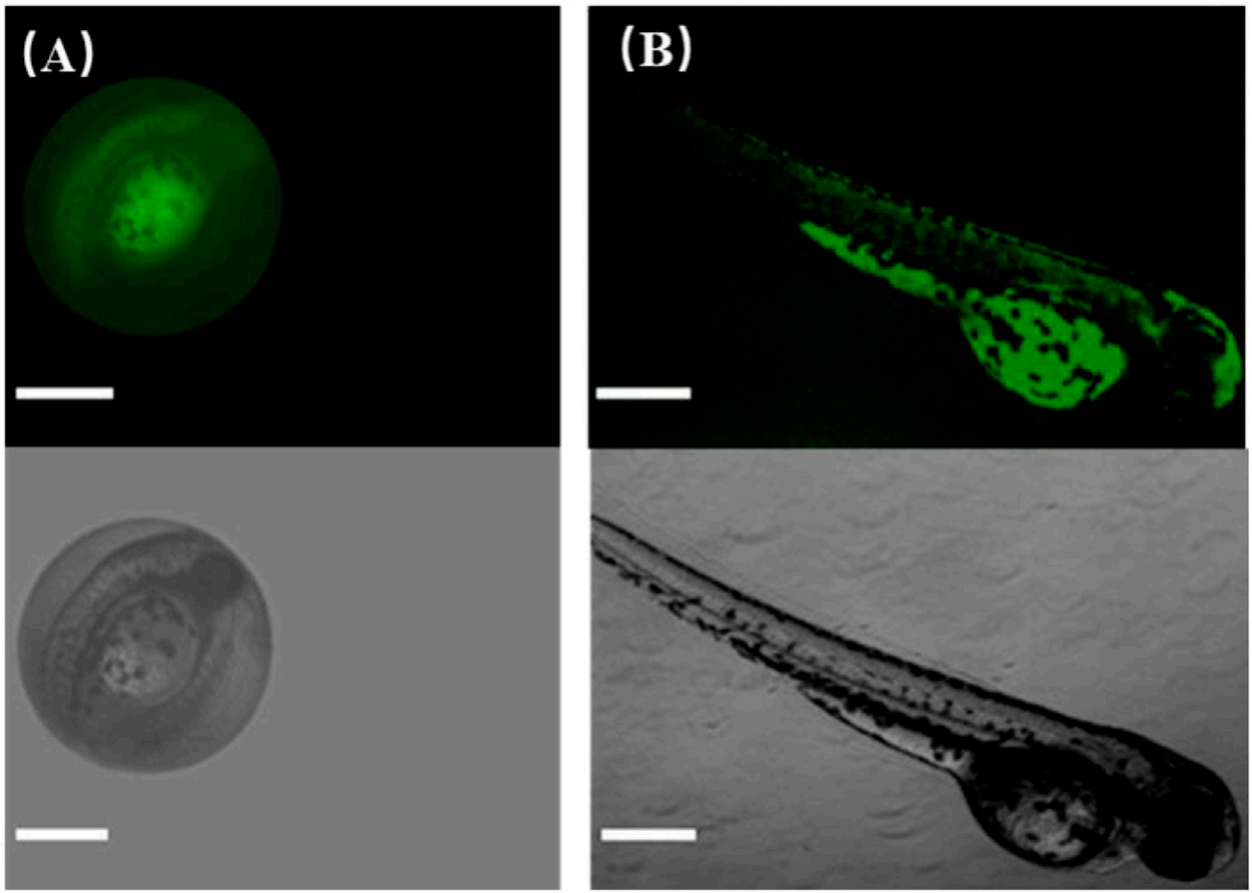
Fluorescence images of CDs in living zebrafish grown for **(A)** 1 day old and **(B)** 5 days old. The scale bar is 500 μm.

Next, to further explore the biological application of CDs, zebrafish was selected as a model to realize fluorescence imaging of CDs because zebrafish and its embryos have been utilized extensively for studying the transport and biocompatibility of nanoparticles and drugs. In our experiments, zebrafish embryos and larvae were imaged by fluorescence microscopy (FITC-channel, Nikon, ECLIPSE Ti2). Figure 8A manifests that zebrafish embryos showed bright green fluorescence after soaking with CDs for 24 h under blue light excitation. The fluorescence brightness between the yolk and the inner of embryos was different because of the different affinities of CDs to these tissues. This phenomenon well validates that CDs could enter into embryos across the chorion and the germ ring through soaking, which mainly accumulated in the yolk sac. Hence, the fluorescence of CDs can observe obviously their distribution in zebrafish embryos, certifying the practicability of CDs as an imaging probe. To better observe the tissue distribution of CDs *in vivo*, the zebrafish larva was used as a model to confirm the imaging application because CDs have been reported to be absorbed and swallowed through the skin into zebrafish. As shown in Figure 8B, the whole-body fluorescence of zebrafish is relatively uniform; especially, the green fluorescence in the yolk sac is obviously stronger than that in other tissues, which is similar to the fluorescence distribution of zebrafish embryos. By the way, CDs in the yolk sac mainly enter the digestive system and can be discharged from the body. It is also confirmed that the brightness of CDs in other tissues, including the tail, can be transmitted to the whole body through the cardiovascular system. Zebrafish experiments have shown that CDs can be transported through the cardiovascular and digestive systems, which has the potential of imaging *in vivo*. The results of zebrafish research can be used in other higher animals because of their high homology with mammals. Thus, CDs are expected to be an effective fluorescent probe for *in vivo* imaging.

### Real Sample Detection

In order to verify the practicability of the probe, we applied it to the detection of the chloride ion in three kinds of real water samples (purified drinking water, river water, and tap water), all of which came from or near the laboratory. The probe solution was configured as CDs (100 µg/ml) with Ag^+^ (2 mM). A standard addition method was used in the whole process. As shown in Table 1, except for the low recovery rate of tap water (93.9%), the recovery rate of other samples is between 97.3% and 103.1, and the RSD is 2–4%, which is satisfactory. The results show that there is no chloride ion in purified water, and the chlorine content in tap water is much higher than that in river water. The real sample analysis shows that the probe can detect the concentration of the chloride ion in an appropriate range.

**TABLE 1:** Result of Cl^−^ determination in real samples.

Sample	Original found(mM)	Added(mM)	Total found(µM)	Recovery(%)	RSD(n = 5, %)
Purified water	0	0.5	0.511	102.2	2.11
	0	1	1.025	102.5	2.23
River water	0.612	0.5	1.147	103.1	2.32
	0.612	1	1.591	98.7	3.07
Tap water	1.311	0.5	1.762	97.3	3.26
	1.311	1	2.17	93.9	4.15

## Conclusion

To sum up, the green CDs synthesized by o-phenylenediamine and L-cysteine can realize the detection of chlorine ions through an “off–on” mechanism. The reasonable detection range is 15–200 μΜ with the LOD as 2.817 μΜ. The competitive reaction confirmed that the CDs with Ag^+^ have a strong selectivity for the detection of chloride ions. Moreover, cellular experiments demonstrated that CDs are safe, non-toxic, and biocompatible, and zebrafish experiments showed that CDs have good potential for fluorescence imaging *in vivo*.

## Data Availability

The original contributions presented in the study are included in the article/Supplementary Material, and further inquiries can be directed to the corresponding authors.
