# Supplementary material for: One-Step Synthesis of Green Fluorescent Carbon Dots for Chloride Detecting and for Bioimaging
Source: Front Chem. 2021 Sep 17;9:718856. doi: 10.3389/fchem.2021.718856 (PMC8484530; doi:10.3389/fchem.2021.718856)
Supplement: Supplementary file 1 [file DataSheet1.PDF]

Supplementary Material

One-Step Synthesis of Green Fluorescent Carbon Dots for Chloride Detecting and for Bio-Imaging

Table S1 Comparison with reported fluorescent Cl^- probes.

Probe	Recognition Mechanism	Linear Range/mM	LOD/mM	Reference
**Ag^+-FBI**	metal coordination	0.1-0.5	0.019	(Kim et al., 2020)
**dicationic receptor 1**	PET quenching	0-6	0.033	(Bazany-Rodríguez et al., 2015)
**g-C_3N_4 nanosheets**	PET quenching	0.1-0.5	0.06	(Zhang et al., 2020)
**BeQ1**	heavy atomic collision quenching mechanism	0.1-30	0.046	(Zhang et al., 2018)
**CA–cysteine**	halide quenching	10-280	0.373	(Kim et al., 2017)
**Cp*Rh(Fz)(H_2O)**	metal coordination	0.1-30	0.3	(Riis-Johannessen et al., 2010)
**this work**	metal coordination	0.2-0.4	0.0028	this work

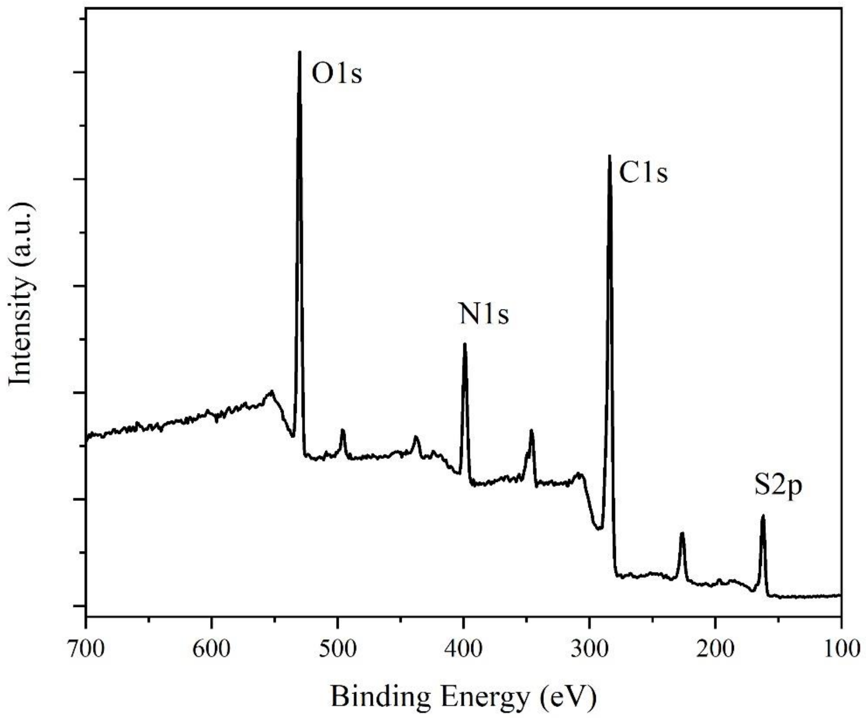

Fig. S1. Full XPS spectra of the CDs.

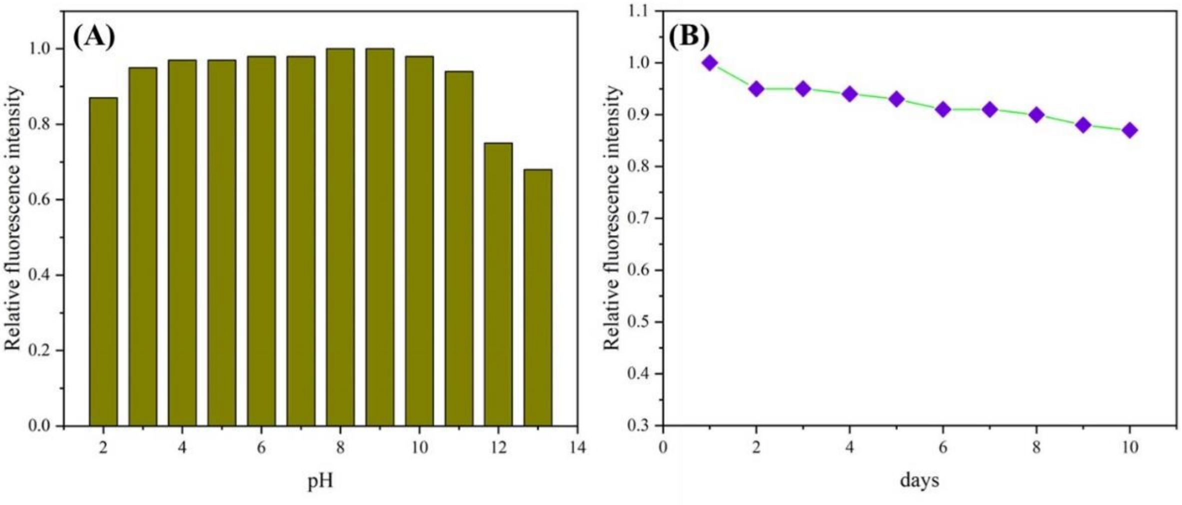

Fig. S2. (A) FL stability of CDs solution under different pH values (from 2 to 13); (B) FL stability of CDs solution after long time storage.

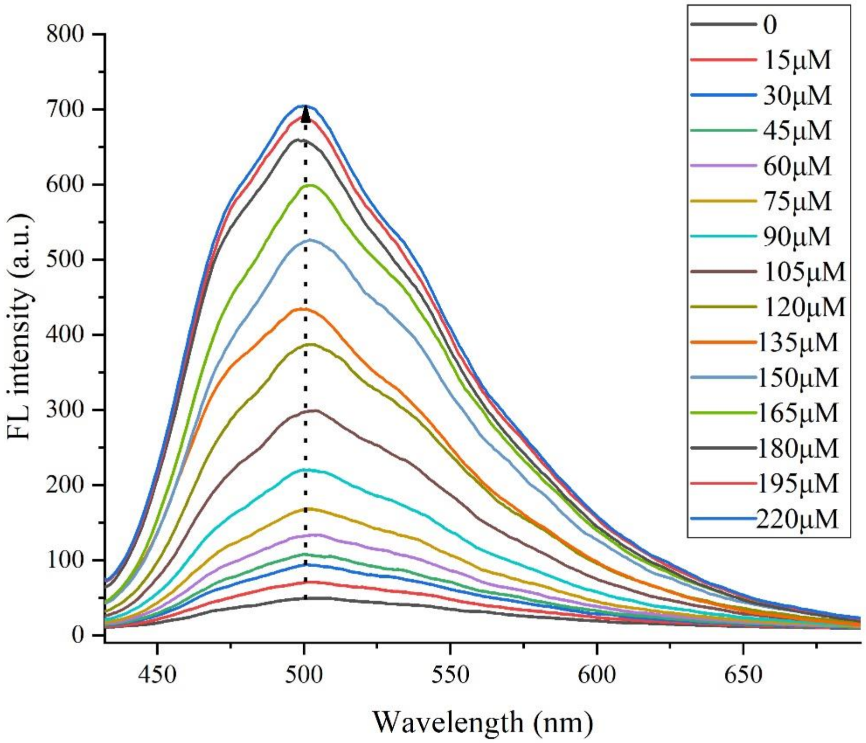

Fig. S3. The fluorescence recovery curves of CDs (5 μg/mL) coating Ag+ (200 μM) with different Cl^- concentrations (from 0 to 225 μM).

Table S2. The fluorescence intensities of CDs (5 μg/mL) coating Ag^+ (200 μM) with different Cl^- concentrations (from 0 to 225 μM).

$c(Cl^-)$	I [a]	$\ln(I)$ [b]
15	49.0	3.89
30	70.9	4.26
45	94.0	4.54
60	107.5	4.68
75	132.7	4.89
90	167.8	5.12
105	220.0	5.39
120	297.5	5.70
135	386.6	5.96
150	434.2	6.07
165	524.7	6.26
180	598.5	6.39
195	657.9	6.49
210	689.4	6.54
225	705.0	6.56

[a] The fluorescence intensity (FL) of the CDs solution.

[b] The natural logarithm of FL values.

LOD calculation

The detection limit (LOD) is defined as LOD = 3σ/S, where σ is the standard deviation of the blank signals and S is the slope of the calibration curve.

In this experiment, σ is calculated to be 0.0139 (the signals of CDs with Ag^+ without Cl^-), and S is 0.0148 (the regression equation: y = 0.0148x+3.853, R^2 = 0.992). Thus, the LOD is calculated as 2.817 μM.

References

Bazany-Rodríguez, I.J., Martínez-Otero, D., Barroso-Flores, J., Yatsimirsky, A.K., and Dorazco-González, A. (2015). Sensitive water-soluble fluorescent chemosensor for chloride based on a bisquinolinium pyridine-dicarboxamide compound. *Sensors Actuators B: Chem.* 221**,** 1348-1355.

Kim, J., Lee, S., Kim, S., Jung, M., Lee, H., and Han, M.S. (2020). Development of a fluorescent chemosensor for chloride ion detection in sweat using Ag+-benzimidazole complexes. *Dyes and Pigments* 177**,** 108291.

Kim, J.P., Xie, Z., Creer, M., Liu, Z., and Yang, J. (2017). Citrate-based fluorescent materials for low-cost chloride sensing in the diagnosis of cystic fibrosis. *Chemical Science* 8**,** 550-558.

Riis-Johannessen, T., Schenk, K., and Severin, K. (2010). Turn-Off-and-On: Chemosensing Ensembles for Sensing Chloride in Water by Fluorescence Spectroscopy. *Inorg. Chem.* 49**,** 9546-9553.

Zhang, F., Ma, C., Wang, Y., Liu, W., Liu, X., and Zhang, H. (2018). Fluorescent probes for chloride ions in biological samples. *Spectrochim. Acta A* 205**,** 428-434.

Zhang, Z., Gao, Y., Li, P., Qu, B., Mu, Z., Liu, Y., Qu, Y., Kong, D., Chang, Q., and Jing, L. (2020). Highly sensitive fluorescence detection of chloride ion in aqueous solution with Ag-modified porous g-C3N4 nanosheets. *Chin. Chem. Lett.* 31**,** 2725-2729.
